# The antibiotic checklist: an observational study of the discrepancy between reported and actually performed checklist items

**DOI:** 10.1186/s12879-017-2878-7

**Published:** 2018-01-08

**Authors:** Frederike V. van Daalen, Marlies E. J. L. Hulscher, Cas Minderhoud, Jan M. Prins, Suzanne E. Geerlings

**Affiliations:** 1 Department of Internal Medicine, Division of Infectious Diseases, Academic Medical Centre, University of Amsterdam, Room F4-132, Meibergdreef 9, 1105 AZ Amsterdam, the Netherlands; 2 Radboud Institute for Health Sciences, Radboud University Medical Center, Nijmegen, the Netherlands

**Keywords:** Checklists, Quality of care, Antibiotic management, Performance measures

## Abstract

**Background:**

Checklists are increasingly used to measure quality of care. Recently we implemented an antibiotic checklist in nine Dutch hospitals and showed that use of the checklist resulted in more appropriate antibiotic use. While more appropriate antibiotic use was associated with a reduction in length of stay, use of the checklist in itself was not. In the current study we explored discrepancies between reported and actually performed checklist items at the patient level to test the validity of checklist answers, to evaluate whether discrepancies between reported and actually performed checklist items could explain the lack of effect of checklist use on length of stay, and to identify missed opportunities for performance per checklist item.

**Methods:**

Checklist answers represented reported performance. Actual performance was assessed by data from the patients’ medical files. Reported and actually performed checklist items could be ‘both YES’; ‘both NO’; ‘YES reported, NOT actually performed’; or ‘NO reported, YES actually performed’. We determined an overall ‘both YES’ score per checklist, and used mixed models to evaluate whether an association existed between this overall score and patient’s length of hospital stay. Finally, we analysed whether the items that were not actually performed, could have been performed.

**Results:**

Between January and October 2015 physicians filled in 1207 checklists. In total 7881 items were checked. Most items were ‘both YES’ (3392/7881, 43.0%) or ‘both NO’ (2601/7881, 33.0%). The number of ‘YES reported, NOT actually performed’ items was 1628/7881 (20.7%) compared to 260/7881 (3.3%) ‘NO reported, YES actually performed’ items. The level of discrepancy between reported and actually performed items differed per checklist item. The item ‘prescribe antibiotic treatment according to the local guideline’ had the highest percentage of ‘YES reported, NOT actually performed’ items, namely 45.1%. A higher overall ‘both YES’ score of the checklist was significantly associated with a shorter length of hospital stay. Of all checklist items 21.8% were not performed while they could have been performed.

**Conclusions:**

Checklist answers do not accurately assess actual provided care. As actual performance of the antibiotic checklist items is associated with length of stay, efforts to increase actual performance appear to be justified.

**Electronic supplementary material:**

The online version of this article (10.1186/s12879-017-2878-7) contains supplementary material, which is available to authorized users.

## Background

Checklists have been embraced in the medical field. Since the impressive success of the WHO surgical safety checklist in 2009 [1], more than 4000 hospitals worldwide implemented modified versions of this checklist [2]. In countries like the UK, Canada and the Netherlands, the Ministry of Health mandates the use of surgical safety checklists. Moreover, some policy makers apply “checklist use” as a measure for quality of care [3, 4].

Previously we developed and introduced an antibiotic checklist to improve the quality of antibiotic use by reminding physicians of the most important steps in recommended appropriate antibiotic use [5, 6]. Such improvement is not only necessary to curb antimicrobial resistance, but has also been associated with various patient outcomes, including reduced length of hospital stay [7–10]. In our study, use of the antibiotic checklist resulted in more appropriate antibiotic use. We found that more appropriate antibiotic use was significantly associated with a shorter length of hospital stay for the patient. However, use of the checklist in itself was not associated with a reduction in length of stay [6]. One probable explanation for these contradictory results could be that ‘completing a checklist’ and ‘actual performance of the checklist items’ are in practice not the same.

Although previous studies have suggested that variation in actual compliance with checklists exists [2, 11, 12], so far this has not been illustrated with data at patient level. In the current study we explored discrepancies between reported and actually performed checklist items at the patient level to test the validity of checklist answers, to evaluate whether discrepancies between reported and actually performed checklist items could explain the lack of effect of checklist use on length of hospital stay in the clinical trial, and to identify missed opportunities for performance per checklist item.

## Methods

### Study design and setting

This observational study was performed alongside a stepped wedge cluster randomised trial evaluating the effectiveness of introducing an antibiotic checklist [6]. Patients were included in two university- and seven teaching hospitals in the Netherlands between January and October 2015. In each hospital at least one surgical, one non-surgical, and the emergency department participated.

### Appropriate antibiotic use – The antibiotic checklist

The antibiotic checklist was meant as a supporting tool to remind physicians of the most important steps in recommended appropriate antibiotic use [6]. It consisted of seven generic quality indicators that define appropriate antibiotic use in the hospital (Additional file 1: Figure S1) [13, 14]. The checklist was divided into two bundles. The first bundle (five items) had to be completed at the moment of prescribing IV antibiotics. The second bundle (two items) had to be used during the course of treatment, at the latest after 72 h of treatment. A barrier survey was performed prior to checklist introduction to optimize its usability in daily practice [5]. One result of this survey was that 17.4% of the physicians thought the checklist was too complex for use in daily practice. Therefore we adapted the checklist by including tick-boxes and pre-printed options. For each item on the checklist it was possible to choose the answer “YES” or “NO”. If “NO” was checked, physicians could indicate the reason for this decision. For example, therapy could not be adapted based on culture result because a positive culture result was lacking.

Physicians completed the checklists for hospitalized adults (≥18 years old) or adults at the emergency department who were admitted at a participating ward with a suspected community-acquired and/or hospital-acquired bacterial infection and were prescribed intravenous antibiotics. The participants were both residents and specialists with different levels of experience. One month preceding the start of checklist use in each hospital, introduction of the antibiotic checklist was prepared. The checklists were displayed in printed form at all working places at the participating departments. Stimulating activities to use the checklist, such as education, feedback and reminders, were organised. It was the physicians’ responsibility to use the checklist each time an antibiotic was started. Physicians were informed that the effect of checklist use on the appropriateness of antibiotic use and on length of hospital stay would be measured. They did not know that their answers on the checklist would be checked against the electronic medical records.

### Assessments

We included checklists used for eligible patients with at least one item checked. [6] The answers ticked on the checklist represented the reported performance of the item at patient level. The arguments for non-performance that were reported by the physician on the completed checklist were defined as ‘reasons for non-performance’.

Actual performance of the checklist items was assessed by collection of data from the patients’ electronic medical records (EMR), including medication charts and laboratory results. Data were recorded on the collection of blood cultures, the collection of cultures of suspected sites of infection, antibiotic use including dosage, route of administration and duration of treatment, relevant laboratory parameters, suspected type of infection and, if relevant, changes in diagnosis.

### Analysis

The ticks on the checklist directly assessed the reported performance. For the actual performance, the data of the patients’ EMRs were evaluated using previously developed algorithms.^14^ For example -concerning the checklist item ‘prescribe antibiotic treatment according to the local guideline’- when a patient was treated with ceftriaxone because of a suspected community-acquired pneumonia, an algorithm determined whether that treatment was according to the local guideline.

Per checklist item we determined the concordance between reported performance and actual performance. We distinguished positive (“YES”) and negative (“NO”) answers. Reported and actually performed checklist items could be:

1. Both YES. E.g., both the checklist answer “YES, I prescribe antibiotic treatment according to the local guideline” and the algorithm indicated that the antibiotic treatment was according to the local guideline;
2. Both NO. E.g., both the checklist answer “NO, I do not prescribe antibiotic treatment according to the local guideline” and the algorithm indicated that the antibiotic treatment was not according to the local guideline;
3. YES reported, NOT actually performed. E.g., the checklist answer “YES, I prescribe antibiotic treatment according to the local guideline” indicated that antibiotics were prescribed according to the guideline, while the algorithm indicated that the antibiotic treatment was not according to the local guideline;
4. NO reported, YES actually performed. E.g., the checklist answer “NO, I do not prescribe antibiotic treatment according to the local guideline” indicated that antibiotics were not prescribed according to the guideline, while the algorithm indicated that the antibiotic treatment was according to the local guideline.

First we determined the validity of the checklist answers. The items ‘YES reported, NOT actually performed’ and ‘NO reported, YES actually performed’ indicated differences between reported and actual performance. Both items disrupted the validity of the reported checklist answers. In case of ‘YES reported, NOT actually performed’ the reported checklist answer gave an overestimation of actually provided care, while in case of ‘NO reported, YES actually performed’ the reported checklist answer gave an underestimation of actually provided care.

Previous studies showed an association between actual performance of the checklist items and a shorter length of hospital stay. [6, 15] In the clinical trial we saw that completing the antibiotic checklist was not associated with a reduction in length of stay [6], suggesting that ‘actual performance of checklist items’ is not the same as ‘checklist completion’. To evaluate whether these differences could explain the lack of effect on length of hospital stay in the clinical trial, we investigated if patients in whom a checklist was used and the items were actually performed had a shorter length of stay compared to patients in whom checklist use did not lead to actual performance of the items. To do so, we calculated an overall ‘both YES’ score per checklist. This score expressed the total number of ‘both YES’ items on that particular checklist (maximum of 7). We distinguished very low (≤1 ‘both YES’ items), low (2 ‘both YES’ items), medium (3 ‘both YES’ items), high (4 ‘both YES’ items) and very high (≥5 ‘both YES’ items) overall scores. We evaluated whether an association existed between the overall ‘both YES’ score and the geometric mean of length of hospital stay of the patients.

For all not actually performed items we determined the applicability to the particular patient using algorithms [14]. A ‘missed opportunity for performance’ was a checklist item that was not actually performed while it could have been performed according to the algorithm, independently of the answer on the checklist. E.g., the item “adapt dosage to renal function” only applied to patients with a declined renal function and treatment with an antibiotic of which dosage adjustment was required. The algorithm took these parameters into account to determine the applicability. When “adapt dosage to renal function” applied to the patient, but the item was not actually performed, a missed opportunity for performance was indicated. We evaluated how often these missed opportunities for performance occurred per checklist item. In this evaluation we distinguished items in which the non-performance was reported (thus, ‘both NO’ items) and items in which the non-performance was not reported (thus, ‘YES reported, NOT actually performed’ items). We also performed an in-depth analysis of all items with a missed opportunity for performance, in which we determined when or why the physicians did not actually perform the item while it could have been performed.

The study was exploratory and therefore we used mainly descriptive measures. Frequencies of ‘both YES’, ‘both NO’, ‘YES reported, NOT actually performed’ and ‘NO reported, YES actually performed’ items were expressed in absolute numbers and percentages. The association between overall ‘both YES’ scores and length of hospital stay was evaluated using generalised linear mixed models. These models account for within-cluster dependencies, which is required as checklists from nine different hospitals were included. Furthermore, the models adjust for possible confounders, which is necessary because in the clinical trial several covariates appeared to influence length of stay. We included the hospitals (clusters) as a random effect, and patient and treatment characteristics -age, comorbidity, type of diagnosis, community vs hospital acquired infection, antibiotics started at the Emergency Department versus ward [6]- as fixed effects in the mixed model analysis. *P* < 0.05 was considered statistically significant. Analyses were done using IBM SPSS Statistics, version 23.0.

## Results

In 1207 of the 5354 eligible patients a checklist was used (22.5%). Of the 1207 included checklists 993 (82.3%) were fully and 214 (17.7%) were partly completed. In total 7881 checklist items were checked.

**Fig. 1:**
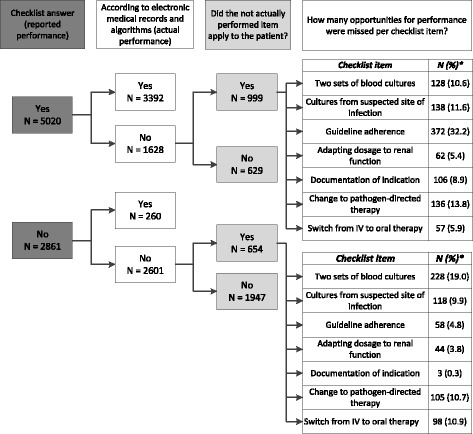
Number of reported and actually performed checklist items

Figure 1 shows the number of reported and actually performed checklist items. Most items were ‘both YES’ (3392/7881, 43.0%) or ‘both NO’ (2601/7881, 33.0%). The number of ‘YES reported, NOT actually performed’ items was 1628/7881 (20.7%) compared to 260/7881 (3.3%) ‘NO reported, YES actually performed’ items. Thus, overall, the reported checklist answers gave an overestimation of the actually provided care.

The level of concordance between reported and actually performed items differed per checklist item (Table 1). Items were most often ‘both YES’ for ‘documentation of indication’ (90.4%), and most often ‘both NO’ for ‘adapt dose to renal function’ (72.9%). The percentage of ‘YES reported, NOT actually performed’ items was remarkably high for ‘prescribe antibiotic treatment according to the local guideline’, namely 45.1%. The percentage of ‘NO reported, YES actually performed’ items was low for all checklist items.

**Table 1:** Concordance per checklist item

Checklist item	Both YES^a^ (% of all answers for this checklist item)	Both NO^a^ (% of all answers for this checklist item)	YES reported, NOT actually performed^a^ (% of all answers for this checklist item)	NO reported, YES actually performed^a^ (% of all answers for this checklist item)	Total number of answers for this checklist item^a^
Blood cultures	784 (65.2)	228 (19.0)	128 (10.6)	62 (5.2)	1202 (100)
Cultures from suspected site of infection	571 (47.9)	417 (35.0)	173 (14.5)	30 (2.5)	1191 (100)
Guideline adherence	483 (40.4)	116 (9.7)	540 (45.1)	58 (4.8)	1197 (100)
Adapt dose to renal function	66 (5.7)	841 (72.9)	223 (19.3)	24 (2.1)	1154 (100)
Documentation of indication	1076 (90.4)	3 (0.3)	106 (8.9)	5 (0.4)	1190 (100)
Adapt therapy when cultures become available	126 (12.8)	556 (56.5)	253 (25.7)	49 (5.0)	984 (100)
IV-oral switch	286 (29.7)	440 (45.7)	205 (21.3)	32 (3.3)	963 (100)

^a^numbers are N (%)

**Fig. 2:**
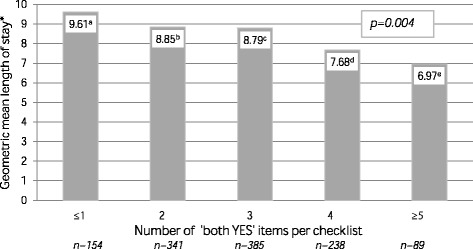
Association between the overall ‘both YES’ scores of the checklists and length of hospital stay. Legends:*after correction for the same covariates as in the clinical trial [6], namely: age, comorbidity, type of diagnosis, community- versus hospital-acquired infection and antibiotics started at the Emergency Department versus ward, ^a^ 95% confidence interval 8.41–11.13, ^b^ 95% confidence interval 7.24–8.67, ^c^ 95% confidence interval 6.62–7.92, ^d^ 95% confidence interval 5.21–6.36, ^e^ 95% confidence interval 4.31–5.70

Figure 2 presents the association between the overall ‘both YES’ scores of checklists and the geometric mean of length of hospital stay of the patients. Patients with more ‘both YES’ items – YES the checklist item was reported and YES the checklist item was actually performed – had a significantly shorter length of hospital stay compared to patients with less ‘both YES’ items on the checklist.

Figure 1 also illustrates how often the items that were not actually performed, could have been performed. In 999/1628 of the ‘YES reported, NOT actually performed’ items (61.4%) the item applied to the patient, indicating that 999 opportunities for performance were missed. Most opportunities for performance were missed for guideline adherence (Fig. 1).

Table 2 presents the most common causes of discrepancy between reported and actual performance in these 999 items. For example, the majority of the patients with a ‘YES reported, NOT actually performed’ item for ‘culture from suspected site of infection’ were diagnosed with a respiratory tract infection (86/138 patients (62%)), whereas only nine patients (7%) were diagnosed with a urinary tract infection.

**Table 2:** Overview of situations in which physicians did not actually perform a checklist item which could have been performed

Checklist item	‘YES reported, NOT actually performed’ while checklist item applied to the patient (N)	Information about actual performance	N (%)
Take at least two sets of blood cultures before starting systemic antibiotic therapy	128	Only one set of blood cultureswas performed	110 (86)
Take specimens for cultures from suspected sites of infection	138	Diagnosis was a respiratory tract infection, sputum culture was not performed	86 (62)
		Two possible diagnoses were recorded, only one culture was performed	35 (25)
		Diagnosis was a urinary tractinfection, urine culture was not performed	9 (7)
Prescribe systemic antibiotic treatment according to the local guideline	372	Antibiotic treatment for a respiratory tract infection not according to the guidelines	123 (33)
		Antibiotic treatment for two diagnoses one or both not according to the guidelines	68 (18)
		Antibiotic treatment for a urinary tract infection not according to the guidelines	64 (17)
		Antibiotic treatment for a skin infection not according to the guidelines	40 (11)
Adapt dose and dosing interval of systemic antibiotics to renal function	62	No adaption while eGFR <10 mL/min	7 (11)
		No adaption while eGFR 10–30 mL/min	37 (60)
		No adaption while eGFR 30–50 mL/min	18 (29)
Document the indication for antibiotic treatment in the case notes or electronic medical record (EMR)	106	No documentation	106 (100)
Adapt therapy when culture results become available	136	Change took place on the fourth day of therapy	34 (25)
		Change took place on the fifth day of therapy	8 (6)
Switch from intravenous to oral antibiotic therapy after 48–72 h	57	Switch was performed on the fourth day of therapy	27 (47)
		Switch was performed on the fifth day of therapy	11 (19)

Of all ‘both NO’ items, 25.1% could have been performed according to the algorithms (654/2601) (Figure 1). The most opportunities for performance were missed on the checklist item ‘take two sets of blood cultures’, namely 19.0% (228/1202).

The reasons physicians reported on the checklists for non-performance in case of ‘both NO’ items are presented in Table 3. This table illustrates that physicians’ opinions can be contrary to the algorithms [14]. For example, in 32 patients physicians reported that switching intravenous to oral treatment could not be performed because of ‘insufficient clinical improvement’, while according to the algorithm – which was based on criteria for a safe early switch –[14, 16], the clinical improvement was sufficient for switching to oral therapy.

**Table 3:** Reported reasons for non-performance of an applicable checklist item

Checklist item	Given argument by the physician who completed the checklist for non-performance of the checklist item	Number
*Take at least two sets of blood cultures before starting systemic antibiotic therapy*	In my opinion, blood culturesare not necessary with this diagnosis	84
	Only one set performed instead of two (without explanation)	45
	No indication for blood culture performance because the patient has no fever	25
	Unclear why blood cultures are not performed by my colleague	24
	Only one set performed at the emergency department	10
	No reason given	10
	Only one set performed because phlebotomy was difficult	7
	No indication for blood culture performance because the antibiotics are prophylactic	6
	No indication for blood culture performance because antibiotic treatment is based on previous culture result >1 week ago	4
	We only perform cultures from suspected site of infection	3
	No time to perform blood cultures because of critical clinical condition of the patient	2
	Other	8
	*Total*	*228*
*Take specimens for cultures from suspected sites of infection*	No culture possible from suspected site of infection	79
	No sputum production with a suspected respiratory tract infection	19
	Not done (without explanation)	5
	Culture performance will follow later	4
	Forgotten to perform cultures from suspected site of infection before start of therapy	3
	No reason given	3
	Other	5
	*Total*	*118*
*Prescribe systemic antibiotic treatment according to the local antibiotic guideline*	No reason given	22
	Following other guidelines	7
	Several possible diagnoses: it is notclear which guideline should be followed	5
	We deviate fromlocal guidelines after consulting microbiologist	4
	Antibiotic treatment is based on previous antibiotic therapy	4
	We deviate fromlocal guidelines because mysupervisor prefers another antibiotic	3
	Gentamycin should be given according to the local guidelines,however we did not prescribe gentamycin since the patient is not septic	2
	Other	11
	*Total*	*58*
Adapt dose and dosing interval of systemic antibiotics to renal function	This quality indicator is not applicable to this patient	38
	No reason given	3
	Peritoneal dialysis	1
	eGFR just below normal: expectation that renal function will improve quickly	1
	Renal function not known	1
	*Total*	*44*
*Document the indicationfor the antibiotictreatment in the case notes or electronic medical record (EMR)*	No reason given	1
	Fever of unknown origin, and thus we do not know what to document	1
	Cefuroxime is started at the emergency department but the indication is not clearly explained	1
	*Total*	*3*
*Adapt therapy when culture results become available*	No culture result (yet)	46
	No reason given	18
	Pathogen is susceptible to the current antibiotic treatment	15
	Treatment based on clinical condition	9
	Several pathogens are cultured: doubts about relevance	4
	Treatment was already started based on culture results	3
	Treatment chosen after consulting microbiologist	2
	Other	8
	*Total*	*105*
*Switch from intravenous to oral antibiotic therapy after 48–72 h*	Insufficient clinical improvement	32
	No oral antibiotic available	16
	No oral therapy possible with this diagnosis	14
	Antibiotic treatment is stopped	8
	Continue IV (without explanation)	7
	Prefer to treat five days intravenously	5
	No adequate oral intake/gastrointestinal absorption	3
	No reason given	4
	Unclear diagnosis and unclear to which antibiotic should be switched	3
	No culture results	3
	After consulting microbiologist	2
	Allergy	1
	*Total*	*98*
*Total*		*654*

## Discussion

In this study we have illustrated a discrepancy between reported and actually performed appropriate antibiotic use as captured by the checklist items of the antibiotic checklist in the hospital. Approximately 21% of all items were reported while not actually performed, giving an overestimation of actually provided care. Patients with more reported and actually performed (‘both YES’) items had a shorter length of hospital stay compared to patients with less ‘both YES’ items. This association is in line with previous results [10, 15], which supports the suggestion that discordance between reported and actual performance has disrupted the impact of the checklist on length of stay. In total 1653 (999 + 654) items were not performed while they could have been performed according to the algorithms, suggesting missed opportunities for performance.

Since 21% of all reported items gave an overestimation of actually provided care, the antibiotic checklist cannot be considered a valid instrument to measure provided quality of care. Although much less detailed than in our study, the existence of discrepancy between reported and actual performance has been described previously [2, 17–20]. For example, the time-out section of the Surgical Safety Checklist was actually performed as intended in 38.5% of the 294 observed operations in the UK while claimed to have been done in more than 95% of these operations by one team member (most often the scrub nurse) at the operation room [17].

According to our algorithms, 1653 opportunities for performance were missed, indicating room for improvement of performance. Several studies reported the room for improvement on performance per checklist item for (parts of) the Surgical Safety Checklist [2, 17–19, 21–23]. This room was considerable and varied between 3 and 100% per checklist item. In our study the room for improvement of performance varied between 9 and 41% per checklist item, with most missed opportunities for prescribing antibiotic treatment according to the local guideline. In the majority of these items performance was reported while not actually done. We do not know whether physicians were aware of the discrepancies between reported and actually performed items. It has been noticed before that the perception of physicians of their performance is higher than actual performance [2, 18, 24]. For example, an analysis of physicians’ perceptions on antimicrobial use illustrated that most physicians agreed that antibiotics are overprescribed, but only a small proportion felt that they themselves overprescribed [24].

The second item with a relatively high percentage of missed opportunities for performance was the performance of two sets of blood cultures. The majority of these items were ‘both NO’ (not reported and not actually performed), meaning that the physician intentionally did not perform blood cultures. The common explanation for non-performance (84 times) was that the physician thought blood culture performance was not necessary with the suspected diagnosis. In our barrier study several physicians already mentioned these doubts, wherefore we added the option “In my opinion, not necessary with this diagnosis” in the checklist [5]. Although in an international Delphi procedure taking blood cultures was considered a quality indicator for appropriate antibiotic use in all hospitalized patients with intravenous antibiotic treatment [6, 13, 14], the clinical relevance of blood cultures in some diagnoses has been questioned [25], and the finding that this answer option was often used in our study, suggests that taking blood cultures might not be agreed-upon standard practice. This should be taken into account for future checklist use, as disagreement about the contents of an intervention makes it very unlikely that it will be successful [26].

Our study has several strengths. To our knowledge, our study is the first to evaluate the discordancy between reported and actually performed checklist items by using the ticks on the box reported by the physician him- or herself at the moment of performance. These data provide very detailed information per checklist item on the physicians’ perception of their own performances. Additionally we provided information about physicians’ considerations for resigning from recommended antibiotic care. We included a large number of checklists collected in nine representative Dutch hospitals. Finally, we illustrated that this in-depth analysis can help to better understand other results, such as, in our study, the lack of effect of checklist use on length of stay. Our findings are very relevant, since checklists are increasingly used to measure quality of care [3, 4], with sometimes consequences for the reimbursement of this care. This study showed that checklist use is not equal to actual performance of checklist items and therefore checklists cannot be considered a valid instrument to measure provided quality of care.

Our study also has limitations. We did not include the overall completion rate in this evaluation. As in the majority of the eligible patients in the clinical trial a checklist was not used (77.5%) [6], it is possible that the (partially) completed checklists were filled in by physicians who were motivated to improve the quality of their antibiotic care, resulting in a certain selection bias. On the other hand, when physicians are forced to fill in checklists, discrepancies may even be higher. Another limitation could be that the actual performance was based on documentation in the EMRs and not on direct observations. However, due to extensive search in all sources available, including the laboratory results and medication charts, we think this data reliably reflects actually performed items.

The general limitation of algorithms is that they can do injustice to the complexity of patient care and clinical judgement [27]. For example, we know from clinical practice that obtaining a sputum culture can be challenging in practice, resulting in more non-performed items despite the intention (YES reported) of performance (Table 2). Likewise, as illustrated in Table 3, sometimes physicians had valid reasons for non-performance of checklist items in our study. For example, deviating from local guidelines after consulting a microbiologist is most likely in favour of patient care. Therefore performance scores of 100% are unrealistic and even undesirable.

In future research the economic perspectives of using the antibiotic checklist should be considered. When the checklist is further implemented, the indicated missed opportunities for performance can guide local antibiotic stewardship teams in designing interventions. For example, 123 items were ‘YES reported, NOT actually performed’ for ‘prescribing antibiotic treatment according to the local guideline’ while the suspected diagnosis was a respiratory infection, and so education about the guidelines for respiratory tract infections could be considered. Likewise, since in 110 of the ‘YES reported, NOT actually performed’ items of ‘two sets of blood cultures’ only one set was performed, the importance of taking two sets of blood cultures should be emphasized [28]. Building the checklist into the EMR could also improve actual performance of checklist items. Integration of the Surgical Safety Checklist in the EMR resulted in a significant increase in checklist compliance [29].

## Conclusion

In conclusion, the antibiotic checklist should be used as a supporting tool, not as an instrument to measure actual provided care. As actual performance of the antibiotic checklist items was associated with the patients’ length of hospital stay, efforts to increase actual performance appear to be justified.

## Additional file

Additional file 1: The antibiotic checklist. (PDF 207 kb)

## Abbreviations

EMR: Electronic Medical Record
