# Supplementary material for: The antibiotic checklist: an observational study of the discrepancy between reported and actually performed checklist items
Source: BMC Infect Dis. 2018 Jan 8;18:16. doi: 10.1186/s12879-017-2878-7 (PMC5759243; doi:10.1186/s12879-017-2878-7)
Supplement: Additional file 1: — The antibiotic checklist. (PDF 207 kb) [file 12879_2017_2878_MOESM1_ESM.pdf]

**Phase 1: the start of antibiotic treatment intravenously**		**Yes**	**No**
1	Take at least two sets of blood cultures before starting systemic antibiotic therapy	☐	☐ In my opinion, not necessary with this diagnosis
			☐ Cultures have been taken < 1 week ago
			☐ …………………………………………………………
2	Take specimens for culture from suspected sites of infection	☐	☐ No culture possible
			☐ Cultures have been taken < 1 week ago
			☐ …………………………………………………………
3	Prescribe systemic antibiotic treatment according to the local antibiotic guideline	☐	☐ According to other guidelines, namely ……………..
			☐ Allergy
			☐ Treatment based on previous culture results
			☐ …………………………………………………………
4	a. Determine renal function	☐	☐ ………………………………………………………....
	b. Adapt dose and dosing interval of systemic antibiotics to renal function	☐	☐ Not applicable
			☐ …………………………………………………………
5	Document the indication for the antibiotic treatment in the case notes or electronic medical record (EMR)	☐	☐ …………………………………………………………
**Phase 2: after 48-72 hours of treatment**		**Yes**	**No**
6	Adapt therapy when culture results become available	☐	☐ ………………………………………………………
	Switch from intravenous to oral antibiotic therapy after 48-72 hours	☐	☐ Insufficient clinical improvement
			☐ No available oral antibiotic
			☐ No adequate oral intake/ gastrointestinal absorption
			☐ No oral therapy possible with this diagnosis ……………
			☐ …………………………………………………………
